# Vestibular Function After Cochlear Implantation in Partial Deafness Treatment

**DOI:** 10.3389/fneur.2021.667055

**Published:** 2021-05-21

**Authors:** Magdalena Sosna-Duranowska, Grazyna Tacikowska, Elzbieta Gos, Anna Krupa, Piotr Henryk Skarzynski, Henryk Skarzynski

**Affiliations:** ^1^Institute of Physiology and Pathology of Hearing, Warsaw, Poland; ^2^Medical University of Warsaw, Warsaw, Poland

**Keywords:** cochlear implantation, vestibular evoked miogenic potentials, round window approach, video head impulse test, caloric test

## Abstract

**Introduction:** Cochlear implantation is a fully accepted method of treating individuals with profound hearing loss. Since the indications for cochlear implantation have broadened and include patients with low-frequency residual hearing, single-sided deafness, or an already implanted ear (meaning bilateral cochlear implantation), the emphasis now needs to be on vestibular protection.

**Materials and Methods:** The research group was made up of 107 patients operated on in the otorhinolaryngosurgery department: 59 females and 48 males, aged 10.4–80.2 years (M = 44.4; SD = 18.4) with hearing loss lasting from 1.4 to 56 years (M = 22.7; SD = 13.5). The patients underwent cVEMP, oVEMP, a caloric test, and vHIT assessment preoperatively, and, postoperatively, cVEMP and oVEMP at 1–3 months and a caloric test and vHIT at 4–6 months.

**Results:** After cochlear implantation, there was postoperative loss of cVEMP in 19.2% of the patients, oVEMP in 17.4%, reduction of caloric response in 11.6%, and postoperative destruction of the lateral, anterior, and posterior semicircular canal as measured with vHIT in 7.1, 3.9, and 4% respectively.

**Conclusions:** Hearing preservation techniques in cochlear implantation are connected with vestibular protection, but the risk of vestibular damage in never totally eliminated. The vestibular preservation is associated with hearing preservation and the relation is statistically significant. Informed consent for cochlear implantation must include information about possible vestibular damage. Since the risk of vestibular damage is appreciable, preoperative otoneurological diagnostics need to be conducted in the following situations: qualification for a second implant, after otosurgery (especially if the opposite ear is to be implanted), having a history of vestibular complaints, and when there are no strict audiological or anatomical indications on which side to operate.

## Introduction

Cochlear implantation (CI) is a well-known method of treating individuals with profound hearing loss. Despite its great effect in restoring hearing, after a CI procedure there is the risk of traumatization of the inner ear causing residual hearing loss or vestibular damage (1–4). Previously, vestibular damage was usually supposed to be negligible, due to the operation of central compensation mechanisms, and was rarely thought to cause persistent disability.

With recent advances in technology, in otosurgical techniques, and in our understanding of hearing electrophysiology, the population eligible for cochlear implantation has been broadened. Not only patients with bilateral profound sensorineural hearing loss but also those with unilateral deafness (5) or partial deafness (6) or the elderly (7) can profit from cochlear implantation. In addition, bilateral implantation in order to achieve better speech discrimination and sound localization is becoming more common (8). This brings new opportunities but also new risks to cochlear implant surgery.

Patients with low-frequency residual hearing (partial deafness) achieve statistically better preoperative results in vestibular tests than do standard implantees, but their vestibular performance may be compromised after a CI procedure (9). Elderly patients are more likely to have comorbidities affecting central compensation mechanisms, for example neurological, orthopedic, psychiatric, or ophthalmological dysfunction. If bilateral vestibular damage should occur, the prognosis is rather poor compared to unilateral dysfunction (10). All these considerations prompt a change of mind toward vestibular preservation and make it important to maintain the labyrinth and vestibulum after a CI procedure.

In the 1990s and into this century, the first steps toward “soft surgery” in cochlear implantation began to be implemented (6, 11, 12). Now the use of soft electrodes, a round window approach (RWA), reduced insertion angles, and use of perioperative steroids has become widespread and has proven to be effective in preserving the cochlear structure (13–15). However, the question of how protective these measures are on the vestibule still remains unanswered.

Many papers have assessed vestibular function following cochlear implantation done via cochleostomy or the round window approach. However, they show a big discrepancy in the incidence of postoperative vestibular deterioration: for cochleostomy, the figures are 31–86% for cervical Vestibular Evoked Myogenic Potentials (cVEMPs), 6–50% for caloric tests, and 4–9% for video Head Impulse Test (vHIT); for the round window approach, the comparable figures are 0–76% for cVEMPs, 5–37% for ocular Vestibular Evoked Myogenic Potentials (oVEMPs), and 0–93% for caloric tests (16–28). Moreover, the effect of electrode type and length on vestibular function remains unclear.

The aim of this study was to assess the safety of cochlear implantation in partial deafness in terms of vestibular preservation, with hearing preservation (HP) techniques and range of electrode types.

**Figure 1:**
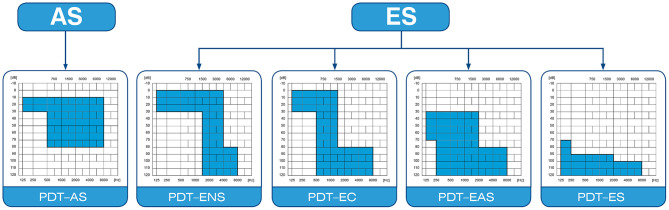
Four categories of partial hearing loss (6, 29, 30).

Figure 1 shows the diversity of audiograms categorized as partial deafness. According to the treatment strategy used, the following groups can be distinguished: electro-natural stimulation (PDT-ENS)—patients with normal or only slightly elevated thresholds in the low- and mid-frequency bands, who need electrical complementation with a very short electrode (16–19 mm); electrical complement (PDT-EC)—patients with normal or only slightly elevated thresholds at low frequencies, who need electrical complementation with short electrodes (20–25 mm) and no amplification at the apex; electro-acoustic stimulation (PDT–EAS)—patients with low- and mid-frequency residual hearing who need amplification from a hearing aid for low frequencies and electrical stimulation from implanted electrode (25–28 mm) for mid and high frequencies; and electrical stimulation (PDT-ES)—patients with non-functional residual hearing who rely fully on electrical stimulation (28–31 mm length electrode) but in whom preservation of cochlear structures is still desirable (6, 29, 30).

## Materials and Methods

In total, 149 patients qualified for PDT-EAS, PDT-EC, or PDT-ES cochlear implantation (Figure 1) were enrolled in this study. Of the 149, there were 13 patients who already had a CI and received a second implant, and four who were implanted twice during the study (they received a CI on both sides sequentially), so that finally 153 ears were operated on.

The exclusion criteria included reimplantation cases and the presence of complete vestibular damage prior to implantation—demonstrated by the absence of cVEMP and oVEMP, areflexia in a caloric test with a slow-component velocity (SCV) <12°/s, or covert or overt saccades in all three planes of the vHIT. Additionally, cVEMP and oVEMP were not performed if there were superior semicircular canal dehiscence syndrome (SSCD), inner ear malformation (including large aqueductus vestibule syndrome LVAS), retrocochlear pathology, central nervous system (CNS) pathology affecting the reflex arc (neurodegenerative disease, demyelinating disease, cerebellar pathology), conductive hearing loss, or highly probable conductive hearing loss. Caloric tests were not done if there was a history of canal wall down tympanoplasty, tympanic membrane perforation, inner ear malformation, or cerebellar pathology. The RWA implantation was carried out according to a six-step procedure for Partial Deafness Treatment (PDT): (1) antrotomy; (2) posterior tympanotomy to allow for visualization of the round window niche; (3) puncture of the round window membrane; (4) insertion of the electrode array, approaching the scala tympani directly through the round window membrane; (5) electrode fixation in the round window niche with fibrin glue (with the membrane partially uncovered to preserve its mobility); and (6) fixation of the device in a well-created in the temporal bone (6). We use soft lateral wall electrodes. Exceptionally, in some non-functional residual hearing (PDT-ES) or borderline PDT-EAS and PDT-ES cases, we may choose the perimodiolar electrode. The study protocol and the informed consent form were approved by the Institutional Bioethics Committee (IFPS:/KB/15/2014). All participants gave their written informed consent for participating in the study and publication of the results with maintained anonymity according to General Data Protection Regulations. The study has been conducted in accordance with the World Medical Association's Declaration of Helsinki from 1964.

### VEMP

Candidates participated in presurgery cVEMP and oVEMP assessment and were retested 1–3 months later with the CI switched off. Both tests were performed on Eclipse software (Interacoustics A/S, Denmark). The patients were stimulated with a 500-Hz tone burst 2:2:2 at 97 dB nHL using an insert tip (31, 32). The impedance at each electrode was <2.5 Ω, and other parameters were a stimulus rate of 5.1 per second and a 10–1,000-Hz bandpass filter.

In cVEMP, patients were asked to turn their head 45° away from the examined ear and to tension the sternocleidomastoid muscle (SCM) at a contraction level of 50–150 μV with the assistance of visual feedback from the software. The right and left electrodes were placed at the midpoint between the termination of the muscle at the mastoid and its origin at the sternum on the right and left sides, respectively, with the vertex electrode situated between the sternoclavicular joints, the ground electrode at the forehead. Averaging of 200 stimulus repetitions was done, and two repeated wave patterns were accepted as a positive response. Results were determined based on the amplitude asymmetry ratio (norm <36%) (33), response latencies (P1, N1), and amplitudes (P1–N1) corrected by dividing by the prestimulus sternocleidomastoid muscle (SCM) contraction level.

Following oVEMP standards, the recording electrodes were placed infraorbitally in the midline of the contralateral eye to the side they refer to, with the ground electrode at the forehead and the vertex on the chin. Signal averaging was increased to 500. The patient was instructed to fix their gaze at a point 35° upward (34). The response was regarded as present if two repeatable patterns were recorded. The results were analyzed based on the latency (N1, P1), amplitude (N1–P1), and interaural amplitude ratio (norm <34%) (35).

### Caloric Tests

During the examination, the patient lay recumbent with the head elevated by 30°. Bilateral caloric stimulation used water at 30°C and 44°C for 30 s into the ear canal with each trial preceded by an 8-min break (VisualEyes BNG of Micromedical Technologies). Unilateral weakness (UW) and slow component velocity (SCV) on both sides before and after cochlear implantation were compared. The degree of canal paresis (UW) was calculated based on Jongkees' formula. A difference of UW > 25% between pre- and postoperative measurements toward the implanted ear was judged as a weakened response. The examination was carried out 4–6 months after the operation.

### Video Head Impulse Test

vHIT was performed using an ICS Impulse type 1085 (GN Otometrics). The patient was seated and asked to focus on a spot 1.5 m away. Then abrupt, unpredictable, small-angle (10–20°) head movements were done in three planes: horizontal, LARP (left anterior–right posterior), and RALP (right anterior–left posterior). In each case, 20 impulses were delivered with a minimum peak head velocity of 150°/s. Normal gain (the quotient of head movement speed and eye movement speed) ranged from 0.6 to 1.2. A gain below 0.6 or the appearance of covert or overt saccades was considered as damage to the particular semicircular canal. The test was conducted preoperatively and 4–6 months postoperatively.

### Hearing Preservation

We measured the hearing preservation 3 and 6 months after cochlear implantation using the following formula (36): HP = [1–(PTApost–PTApre)/(PTAmax–PTApre)]^*^100%, where PTApre is the pure tone average measured preoperatively, PTApost is the pure tone average measured postoperatively, and PTAmax is the maximum level generated by the audiometer. The HP (hearing preservation) values were divided into total loss of hearing (no detectable hearing), minimal HP (range 1–25%), partial HP (26–75%), and complete HP (>75%) (36).

### Statistical Analysis

Statistical analysis was performed using IBM SPSS Statistics v.24. A Mann–Whitney *U*-test was used to examine the relationship between age and postoperative vestibular preservation as well as between hearing preservation (in percent) and vestibular preservation. A Chi-square test was used to investigate the relation between sex, type of electrode, its length, and the postoperative test results and the relation between the postoperative affiliation of the HP group and postoperative vestibular function. A paired-sample *t*-test was applied to assess the latency of the VEMP before and after cochlear implantation. In all cases, *p* <0.05 was considered statistically significant.

## Results

From the initially enrolled group of 149 patients (153 ears), 32 patients (34 ears) were excluded due to complete damage of the vestibulum before implantation according to the criteria described in “Material and methods.” The study did not include people who had inner ear malformation (bilateral large vestibular aqueduct syndrome, LVAS, *n* = 1; or an incomplete partition, *n* = 1) or those with other factors affecting the postoperative function of the labyrinth: recurrent vertigo attacks due to possible delayed Meniere's disease within 9 months after cochlear implantation, *n* = 1; the need for reimplantation due to inflammation of the implant bed, *n* = 1; head injury within a few months after cochlear implantation, *n* = 1; the need for reimplantation due to failure of the internal part of the implant, *n* = 1; and Meniere's disease on the implanted side existing preoperatively and active postoperatively, *n* = 1. Additionally, subjects were excluded after a non-standard course of the CI procedure: traumatic electrode insertion (*n* = 2), with the need to apply a trial electrode in one patient, and a narrow round window niche demanding an extended round window approach (*n* = 2).

The final group included 107 patients operated on in the otorhinolaryngosurgery department: 59 females, 48 males, 10.4–80.2 y.o. (M = 44.4, SD = 18.4) with hearing loss lasting from 1.42 to 56 years (M = 22.7; SD = 13.5). The implanted ear was right in 56 cases and left in 51.

Of the 107 patients, 103 were implanted with soft lateral wall electrodes and four with precurved electrodes. Among the 103 patients implanted with soft electrodes, 80 were implanted with ultrasoft Flex electrodes. That is, in terms of inserted electrodes, there were three groups: precurved (*n* = 4), soft/straight (*n* = 23), and ultrasoft (*n* = 80). The range of inserted electrodes included Advanced Bionics HiRes 90k Advantage Mid-scala (*n* = 4), Cochlear Nucleus CI422 (*n* = 5), Cochlear Nucleus CI522 (*n* = 4), Med-El Sonata Standard (*n* = 6), Advanced Bionics HiRes Hi Focus Slim J (*n* = 1), Med-El Sonata Medium (*n* = 3), Med-El Concerto Medium (*n* = 3), Med-El Sonata Form24 (*n* = 1), Med-El Concerto Form24 (*n* = 1), Med-El Sonata Compressed (*n* = 1), Med-El Sonata Flex soft (*n* = 10), Med-El Concerto Flex soft (*n* = 2), Med-El Concerto Flex28 (*n* = 8), Med-El Sonata Flex28 (*n* = 20), Med-El Synchrony Flex28 (*n* = 3), Med-El Sonata_Ti100_ Flex28 (*n* = 1), Med-El Concerto Flex24 (*n* = 8), Med-El Sonata Flex24 (*n* = 17), Med-El Synchrony Flex24 (*n* = 2), Med-El Concerto Flex20 (*n* = 5), and Med-El Sonata Flex20 (*n* = 2).

The tests performed included cVEMP (*n* = 103), oVEMP (*n* = 69), caloric test (*n* = 43), vHIT horizontal semicircular canal (*n* = 28), vHIT anterior semicircular canal (*n* = 26), and vHIT posterior semicircular canal (*n* = 25).

### cVEMP

Of the 103 people who underwent a preoperative examination, responses were recorded in 73 cases. We found a postoperative loss of cVEMPs in 14 of 73 patients (19.2%). The preoperative and postoperative latency of P1 and N1 peak did not differ significantly (*p* = 0.410 and *p* = 0.157, respectively). The rate of saccular loss was not affected by sex (*p* = 0.554). However, it depended on age (Mann–Whitney *U*-test: *U* = 230; *p* = 0.010). A preserved cVEMP response was present in 31 women and 28 men aged 10.4 to 68.2 y.o. (M = 36.2, SD = 17.0) and a lost cVEMP response by nine women and five men aged 30–67.3 years (M = 48.6, SD = 11.4). The causes of hearing loss in patients with an abnormal cVEMP response postoperatively were head injuries (*n* = 1; 1.4%), autoimmune inner ear disease (*n* = 1; 1.4%), sudden idiopathic deafness (*n* = 3; 4.1%), viral infection (*n* = 2; 2.7%), and unknown origin (*n* = 7; 9.6%). The etiology of hearing loss in patients with a preserved VEMP response was much wider: acoustic trauma (*n* = 1; 1.4%) cholesteatoma (*n* = 1; 1.4%), TORCH infection (*n* = 2; 2.7%), genetic mutation (*n* = 2; 2.7%), head injury (*n* = 1; 1.4%), effect of ototoxic drugs (*n* = 4; 5.5%), prematurity (*n* = 4; 5.5%), barotrauma (*n* = 1; 1.4%), sudden idiopathic deafness (*n* = 6; 8.2%), unknown origin (*n* = 35; 48.0%), and viral infection (*n* = 2; 2.7%). Due to the wide diversity of hearing loss causes, no statistical analysis of its effect on test results was undertaken. No statistically significant differences were found regarding the effect of electrode type on postoperative vestibular function (perimodiolar vs. straight vs. ultrasoft, *p* = 0.097), although the incidence of saccular damage was lowest in the group of ultrasoft electrodes. In the three groups implanted with the ultraflex, straight, and precurved electrodes, elicitable cVEMPs were found postoperatively in 49 of 57 patients (86.0%), 9 of 14 patients (64.3%), and 1 of 2 patients (50%), respectively. In a further analysis of the effect of electrode length on postoperative cVEMP responses, two patients with incomplete electrode insertion were excluded. Maintenance of saccular responses was seen in 4/6 (66.7%) using 20-mm electrodes, 23/27 (85.2%) using 24-mm electrodes (Flex 24, Form, Medium), 18/20 (90%) using 28-mm electrodes, and 8/11 (72.7%) using 31-mm electrodes (Flex soft, Standard). A similar analysis restricted to the four subgroups of the Flex group (Flex 20, Flex 24, Flex 28, and Flex soft) showed retained cVEMP in 4/6 patients (66.7%), 19/21 patients (90.5%), 18/20 patients (90%), and 6/8 patients (75%), respectively. There was no significant effect of electrode length either by analyzing within the Flex electrodes (*p* = 0.367) or by pairing different types of lateral wall electrodes into groups of the same length (*p* = 0.437). All the postoperative cVEMP results are summarized in Table 1.

**Table 1:** Postoperative cVEMP results.

**cVEMP test result**		**Present**	**Absent**
Demographic information	Sex (female:male ratio)	31:28	9:5
	Average age (std. deviation)	36.15 (SD = 17.01)	48.57 (SD = 11.35)
Type of electrode	Perimodiolar	1 (50)	1 (50)
	Straight/soft	9 (64.3)	5 (35.7)
	Ultrasoft	49 (86)	8 (14)
Length of electrode^*^	Flex 20	4 (66.7)	2 (33.3)
	Flex 24, Form 24, Medium	23 (85.2)	4 (14.8)
	Flex 28	18 (90)	2 (10)
	Flex Soft, Standard	8 (72.7)	3 (27.3)

*Figures in brackets for the type and length of electrode are percentages*.

* *Two patients with incomplete electrode insertion were excluded*.

### oVEMP

Among the 69 oVEMP tests performed on the patients preoperatively, positive responses were recorded in 46 of them. Of the 46 patients, postoperative losses were found in 8 (17.4%). The difference between preoperative and postoperative N1 and P1 latency was not statistically significant (*p* = 0.066 and *p* = 0.074, correspondingly). The loss of response was not influenced by gender (*p* = 0.999) or age (*U* = 114.00; *p* = 0.271), although the mean age of people with oVEMP loss was higher than the mean age of people with preserved oVEMP responses, and the youngest person with loss of utricular function was 34 years old.

oVEMP responses were preserved in 21 women and 17 men, while no response was recorded in four women and four men. The age of the patients with retained oVEMPs ranged from 11.08 to 68.17 y.o. (M = 40.29, SD = 17.52), and the age of those with newly postoperative absent oVEMPs ranged from 34.50 to 64.25 y.o. (M = 48.91, SD = 10.09). Among patients with oVEMP loss, the etiology of hearing loss was unknown (*n* = 5, 14.9%), head injury (*n* = 1; 2.2%), idiopathic sudden deafness (*n* = 1; 2.2%), and autoimmune inner ear disease (*n* = 1; 2.2%). Patients with recorded postoperative utricular responses had the following hearing loss etiology: acoustic trauma (*n* = 1; 2.2%), cholesteatoma (*n* = 3; 6.5%), TORCH infection (*n* = 1; 2.2%), genetic defect (*n* = 1; 2.2%), ototoxic drugs (*n* = 1; 2.2%), post labyrinthitis (*n* = 1; 2.2%), sudden idiopathic deafness (*n* = 7; 13.0%), viral infection (*n* = 2; 4.4%), and unknown (*n* = 21; 45.7%). Due to the multiplicity of etiological factors, their effect on oVEMP responses after CI was not analyzed.

In terms of the impact of electrode type (precurved, straight, ultrasoft) on postoperative oVEMPs, we found retained responses in one of two (50.0%), eight of nine (88.9%), and 29 of 35 (82.9%), respectively. There was no significant correlation between the frequency of oVEMP loss and the type of electrode (*p* = 0.421). When considering the effect of electrode length on the maintenance of oVEMP responses, two patients with incomplete electrode insertion were excluded from the calculations. The results for preserved oVEMPs after CI were 3 out of 4 (75%) for 20 mm, 18 out of 20 (90%) for 24 mm, 9 out of 12 for 28 mm (75%), and 5 out of 5 (100%) for the 31-mm electrode length recipients. If one only takes into consideration electrode length within the Flex group, the percentage of postoperatively recorded oVEMPs was 75% (3 out of 4) for the Flex 20, 86.7% (13 out of 15) for the Flex 24, 75% (9 out of 12) for the Flex 28, and 100% (3 out of 3) for the Flex soft group. Similarly to the cVEMP responses, no statistically significant relationship was found between electrode length and the risk of possible postoperative oVEMP loss, either for the Flex electrodes alone (*p* = 0.698) or when comparing groups of electrodes of the same length (*p* = 0.462). The postoperative prevalence of oVEMPs is shown in Table 2.

**Table 2:** Postoperative oVEMP results.

**oVEMP test result**		**Present**	**Absent**
Demographic information	Sex (female:male ratio)	21:17	4:4
	Average age (std. deviation)	40.29 (SD = 17.52)	48.91 (SD = 10.09)
Type of electrode	Perimodiolar	1 (50.0)	1 (50.0)
	Soft	8 (88.9)	1 (11.1)
	Ultrasoft	29 (82.9)	6 (17.1)
Length of electrode^*^	Flex 20	3 (75.0)	1 (25)
	Flex 24, Form 24, Medium	18 (90.0)	2 (20.0)
	Flex 28	9 (75.0)	3 (25.0)
	Flex Soft, Standard	5 (100)	0 (0)

*Numbers in brackets for the type and length of electrode are in percentage*.

* *Two patients with incomplete electrode insertion were excluded*.

### cVEMP vs. oVEMP

Altogether, 43 patients elicited both cVEMP and oVEMP responses preoperatively.

Of the 34 subjects with a preserved cVEMP response postoperatively, all presented oVEMP responses within the normal range. Of the nine with a lost cVEMP response after CI, six lost the oVEMP response and three retained it. However, in two of three people with a preserved oVEMP response, there was a significant change in the amplitude asymmetry ratio (by 0.58 and 0.47) with the weakness on the implanted side, and in one other there was only a slight change in this index (0.19) with a correct value in the postoperative examination (0.30 with a predominance of the implanted side). Among 37 subjects with a preserved oVEMP response after CI, 34 presented cVEMP responses simultaneously and 3 patients lost cVEMPs. All six subjects with postoperative oVEMP loss did present cVEMP loss. In summary, maintenance of the cVEMP response (an indicator of saccule function) was always associated with a preserved oVEMP response (which assesses utricle function), whereas loss of oVEMP response was always associated with a loss of cVEMP response.

### Caloric Test

The caloric test was performed pre- and postoperatively in 43 patients, five of whom (11.6%) had a postoperative change in unilateral weakness UW >25% toward the implanted ear.

The group with maintained caloric response consisted of 20 females and 18 males aged 12.3–80.2 years (M = 49.5, SD = 17.8), and those with a weakened response after CI were represented by three females and two males aged 26.0 to 74.8 years (M = 55.4, SD = 18.6). The results were not affected by the age of the patients according to a Mann–Whitney *U*-test (*U* = 75, *p* = 0.449). Due to the small size of the group with weakened responses in the caloric sample, no further statistical analysis was undertaken. The CI recipients with weakened caloric response were implanted with Flex28 (*n* = 2), Flex28 (*n* = 1), Flex soft (*n* = 1), and Medium (*n* = 1), and their hearing losses were caused by ototoxic drugs (*n* = 1), Meniere's disease (*n* = 1), viral infection (*n* = 1), sudden idiopathic deafness (*n* = 1), and unknown factor (*n* = 1). Patients with preserved caloric responses received the following electrodes: CI 422 (*n* = 2; 4.7%), CI 522 (*n* = 1; 2.3%), Compressed (*n* = 1; 2.3%), Flex 20 (*n* = 4; 9.3%), Flex 24 (*n* = 8; 18.6%), Flex 28 (*n* = 12; 27.9%), Form 24 (*n* = 1; 2.3%), Medium (*n* = 2; 4.7%), Mid-scala (*n* = 4; 9.3%), Standard (*n* = 2; 4.7%), and SlimJ (*n* = 1; 2.3%). Their hearing loss etiology was as follows: idiopathic sudden deafness (*n* = 3; 4.7%), acoustic trauma (*n* = 2; 2.3%), cholesteatoma (*n* = 1; 2.3%), genetic (*n* = 1; 2.3%), head trauma (*n* = 1; 2.3%), otosclerosis (*n* = 3; 7.0%), postinflammatory (*n* = 2, 4.7%), Meniere's disease (*n* = 1; 2.3%), autoimmune inner ear disease (*n* = 1; 2.3%), and unknown (*n* = 22; 51.2%).

Among five subjects with weakened caloric responses postoperatively, two of them also showed a loss of cVEMP response, in two cVEMPs were not done, and one patient had a preserved response (with a change in the amplitude asymmetry index by 0.36 with weakness of the implanted ear). oVEMP response in the group with weakened caloric response was as follows: absent in one patient postoperatively, absent in one patient already preoperatively, preserved in one patient (with a change in the amplitude asymmetry index of 0.59 showing weakness of the implanted side), and two patients not tested. The results of caloric tests after CI are shown in Table 3.

**Table 3:** Caloric test postoperative results.

**Caloric test result**		**Normal**	**Weakness**
Demographic information	Sex (female:male ratio)	20:18	3:2
	Average age (std. deviation)	49 (SD = 17.8)	55 (SD = 18.6)
Type of electrode	Perimodiolar	4 (100)	0 (0)
	Soft	10 (90.9)	1 (9.1)
	Ultrasoft	24 (85.71)	4 (14.3)

### vHIT

Postoperative damage to the lateral semicircular canal was found in 2 of 28 patients (7.1%), the anterior semicircular canal in 1 of 26 (3.9%), and the posterior semicircular canal in 1 of 25 (4.0%) patients. One of the patients had damage to all semicircular canals (64.8 y.o. male, RWA, Flex 28). The second patient lost function in the lateral canal, while the anterior and posterior canals presented responses within the normal range (61.7 y.o. female, RWA, Flex soft).

vHIT was preserved in 15 women and 11 men, aged 12.3 to 77.3 years (M = 49.8, SD = 17.7).

### Hearing Preservation

Hearing preservation (HP) was assessed 3 and 6 months postoperatively in 79 CI recipients who had significant preoperative low-frequency residual hearing and so had undergone PDT-EC and PDT-EAS cochlear implantation. cVEMP and oVEMP results were compared with HP at 3 months and with the caloric test, and vHIT results were compared with HP at 6 months after CI, matching the timeline of vestibular tests. Of 10 patients who had postoperative loss of cVEMP responses, hearing preservation ranged from 0 to 100% (M = 48.1%, SD = 42.9) and was described as complete HP (*n* = 3; 30%), partial HP (*n* = 3; 30%), or total hearing loss (*n* = 4; 40%). There were 49 patients with retained cVEMPs who presented HP of 31–100% (M = 79.9%, SD = 19.9) and were classified as partial HP (*n* = 17; 34.7%) or complete HP (*n* = 32; 65.3%). The difference in HP between both groups (lost vs. maintained cVEMPs) was statistically significant in both percentage of preserved hearing (*U* = 146; *p* = 0.042) and affiliation to the particular group (*p* = 0.001). There were six people who lost oVEMPs after CI who had preserved hearing postoperatively consisting of two patients (33.3%) with complete HP, one patient (16.7%) with partial HP, and three patients (50%) with hearing loss (M = 43.9%, SD = 47.4%). Maintained postoperative oVEMP responses (*n* = 33) together with hearing preservation ranged from 31 to 100% (M = 82.7%, SD = 20.5%), and there were 25 CI recipients (75.8%) with complete HP and eight (24.2%) with partial HP. The difference in HP between the patients with and without maintained oVEMPs postoperatively was on the border of statistical significance (*U* = 50.5, *p* = 0.054) and statistical significance (*p* < 0.001) if one considers the percentage and affiliation to each group, respectively. In case of the caloric tests, three patients with weakened responses after CI achieved hearing preservation (0, 55.8, and 60%) 6 months postoperatively (M = 38.6%, SD = 33.5%) and were consequently classified as partial HP (*n* = 2; 66.7%) or total hearing loss (*n* = 1; 33.3%). In contrast, among 25 people with maintained postoperatively caloric responses, hearing preservation was 0 to 100% (M = 72.7%, SD = 26.1%), and so 10 patients (41.7%) were classified as having complete HP, 12 patients (50%) with partial HP, and two patients (8.4%) with total hearing loss. Due to the small numbers, a statistical analysis was not undertaken.

In one case of lateral, anterior, and posterior semicircular canal loss in vHIT, hearing loss of 0% was measured. However, in the group of 16 patients with correct vHIT in the horizontal plane after CI, it was found that seven (43.8%) had complete HP, five (31.3%) had partial HP, and four (25.0%) had total hearing loss (HP ranged from 0 to 100% with M = 58.3 and SD = 36.1). Similarly, in 15 cases of preserved vHIT for the anterior semicircular canal after CI, HP ranged from 0 to 100% (M = 62.2, SD = 33.8) with three patients (20.0%) having total hearing loss, seven (46.7%) with complete HP, and five (33.3%) with partial HP. Finally, the group with correct postoperative vHIT responses for the posterior semicircular canal (*n* = 14) was characterized by HP of 0 to 100% (M = 63.5%, SD = 34.7) and their group affiliations were eight complete HP (57.1%), four partial HP (28.6%), and three total hearing loss (21.4%).

## Discussion

Much research has been done on assessing vestibular function after cochlear implantation surgery, looking for differences in surgical techniques and approaches (in particular, cochleostomy vs. the round window approach) (16–28). A review of the literature does not actually give a clear answer to which access route is better in terms of vestibular preservation. Even trying to define the incidence of vestibular damage after cochlear implantation encounters problems.

In addressing the problem of vestibular damage after a CI, there is first a need to define the criteria of how to analyze and compare otoneurological tests (cVEMP, oVEMP, caloric response, and vHIT) pre- and postoperatively, since there is a definite lack of uniformity in the literature. These criteria should specify which change in response represents definite vestibular damage rather than just say that the test is within or beyond norms.

In the case of the caloric test, the slow component velocity (SCV) may depend on many factors such as the patient's alertness or small differences in performing the exam. It is possible that day-to-day changes in this parameter can be observed even when there are no vestibular changes. UW (unilateral weakness) is a much more reliable parameter to compare. Nevertheless, only specifying a change between categories (normal vs. hyporeflexia vs. areflexia) may falsely lead one to say that there is vestibular damage among patients with borderline Unilateral Weakness (UW), even though the UW change is not significant. Proctor et al. and Piker et al. investigated the minimum detectable change in UW in test–retest exams and found that it was 24% (37) and 23% (38), respectively. It therefore seems reasonable to take a change of UW ≥25% as a marker of lateral semicircular canal damage.

Interpreting vHIT exams is more clear-cut, and detecting new overt or covert saccades in the postoperative period, or drop in the gain of head movement/eye movement to <0.6, should be recognized as vestibular damage. However, the sensitivity of vHIT is a matter for further research and many papers indicate that, among patients with vestibular neuritis or other symptoms suggestive of impairment, a lower percentage have vestibular damage detected by vHIT than by the caloric test (39–41). The same discrepancy was observed in our study. Despite its high specificity, vHIT may not be ideal for identifying minor changes within the vestibule after a CI.

cVEMP and oVEMP are thought to be the most sensitive tools to detect post-CI changes in the vestibulum as they represent the most fragile organs, the saccule and the utricle. The loss of cVEMPs or oVEMPs should be treated as vestibular damage unless there is conductive hearing loss. However, vestibular damage may lead not only to total loss but also to a decrease in amplitude, making analysis more complicated. Comparing the corrected P1–N1 amplitude in cVEMPs, or N1–P1 amplitude in oVEMPs, between pre- and postoperative exams may be erroneous, although some good test–retest reliability has been reported (42). No strict threshold for vestibular damage has yet been identified in terms of change in amplitude or amplitude asymmetry ratio. Elevated thresholds for eliciting VEMPs after a CI procedure may be a good marker of otolith hypofunction. It is worth mentioning that measuring a VEMP threshold extends recording time and necessitates stimulating the ear with multiple high-intensity sounds.

Our study has shown that, based on a wide range of electrodes, partial deafness treatment is protective in terms of vestibular preservation. However, the risk of postoperative vestibular damage is not eliminated. It gives a rate of saccular damage of 19.2% and utricular damage of 17.4% measured by VEMP loss. A reduction in horizontal semicircular canal response was noticed in 11.6% if measured by the caloric response, and damage to the horizontal, anterior, and posterior semicircular canals, as measured by vHIT, of 7.1, 3.9, and 4%, respectively. Hearing preservation was associated with maintenance of vestibular function, and the relation was statistically significant. Patients with elicitable VEMP responses after a CI had at least partially preserved hearing, but never total hearing loss. Weakened caloric tests postoperatively were always associated with at least partial hearing loss.

To properly discuss the counseling of CI candidates, certain facts about central compensation of the unilateral and bilateral vestibular damage need to be recalled. Unilateral vestibular damage can be treated with vestibular rehabilitation including Cawthorne–Cooksey exercises, optokinetic training, virtual reality games, or posturographic training. Such exercises are effective and end with full recovery unless additional comorbidities exist (neurological, psychiatric, orthopedic, ophthalmologic). With bilateral vestibular damage, many functions are affected: postural stability, visual stability during head movements, autonomic cardiovascular reaction of the lower part of the body while standing, cognitive abilities like spatial orientation, navigation abilities, and impairment in dual tasking (10, 42). Only 50% of patients with bilateral hypofunction profit from vestibular rehabilitation (10). In addition, balance may get worse with age and sudden falls may occur. Some symptoms can be easily relieved by rehabilitation exercises like postural stability on an even ground and autonomic vessel reactions in an upright position. Others, like the vestibulo-ocular reflex, can only be partly compensated for by the cervico-ocular reflex or predictive saccades, with handicaps remaining in response to abrupt, unpredictable head movements (43–45). For these reasons, it is reasonable to recommend caution and to consider the potential audiological benefits when deciding to give a second implant in the only-functioning or better vestibulum. The increasing interest in a vestibular prosthesis (46, 47) and vestibulocochlear implants (48) may change our attitude toward bilateral loss of vestibular input. However, as long as such efforts are still under development, and restricted to single clinics and small groups of patients (46–49), we should avoid bilateral vestibular loss.

Our PDT implantation strategy involves applying “soft surgery”: the use of a round window approach via scala tympani which lowers the risk of misinsertion, the administration of postoperative steroids, micropuncture of the round window membrane, insertion of soft electrodes, and reduced insertion angles.

Histological studies have found that vestibular damage is significantly reduced when the electrode is inserted into scala tympani (3, 49). Temporal bone studies indicate that the scala height at the central and lateral portions of scala tympani decreases with increasing distance from the round window (with significant reduction after 450°), whereas the height of the modiolar area remains nearly constant. This increases the risk of unwanted contact of the electrode with the basilar membrane, spiral ligament, or the osseous spiral lamina and consequently the risk of intracochlear trauma. Also, the mechanical properties of the basilar membrane are different depending on the distance from the round window, while the thickness of this structure decreases toward the apex (50–52).

To avoid intracochlear trauma by deep electrode insertion, a flex electrode is used. It has special features such as the five most apical electrode contacts being single, whereas the basal seven electrodes are paired, reducing the diameter of the electrode tip.

Despite the above anatomical facts, we did not find any strong relationship between either electrode type or length and postoperative vestibular function. However, the multiple types of electrodes used restrict the statistical power of being able to see the effect of electrode type on the incidence of vestibular damage. This also agrees with other reports. Nordfalk et al. (22) measured a loss of VEMP responses in five of 14 patients (35.7%) and weakened caloric responses in four out of 10 patients (40%) implanted with a Flex 28 electrode via a round window approach, but, due to the small number of patients, they did not discuss the results of inserting shorter electrodes. Louza and colleagues (25) did not find any statistically significant relationship between postoperative vestibular function and the insertion depth of the electrode (276–707°).

## Conclusions

Hearing preservation techniques in cochlear implantation are connected with vestibular protection, but the risk of vestibular damage is never totally eliminated. The vestibular preservation is associated with hearing preservation, and the relation is statistically significant. Special care and counseling are recommended when qualifying a patient for implantation when that ear has the only (or better) vestibulum, since there is then the risk of bilateral hypofunction or areflexia. Similarly, caution is needed for a patient with comorbidities affecting central nervous system compensation. Therefore, preoperative otoneurological diagnostics are necessary in the following situations: qualification for a second implant, after otosurgery (especially if the opposite ear is to be implanted), with a history of vestibular complaints, with comorbidities that may result in impairment of central compensation mechanisms, and in those who do not have any strict audiological and anatomical indication about which side to operate.

## Data Availability Statement

The datasets generated for this article are not readily available because the patients did not agree to share their data publicly. The datasets are stored on an internal server and are only available to co-workers. All data related to people in the European Union (EU) is protected by law of the General Data Protection Regulation (GDPR). Requests to access the datasets should be directed to m.sosna@ifps.org.pl.

## Ethics Statement

The studies involving human participants were reviewed and approved by Institutional board's ethics committee–Institute of Physiology and Pathology of Hearing, Warsaw, Poland. Written informed consent to participate in this study was provided by each participant or the participants' legal guardian (Only by legal quardian, not next of kin).

## Author Contributions

MS-D: study design, data collection, data interpretation, preparation of manuscript, literature analysis. GT: data interpretation, preparation of manuscript. EG: data analysis/statistics. AK: data collection, preparation of manuscript. PS and HS: study design, data collection. All authors contributed to the article and approved the submitted version.

## Conflict of Interest

The authors declare that the research was conducted in the absence of any commercial or financial relationships that could be construed as a potential conflict of interest.
